# Folfirinox versus gemcitabine-cisplatin combination as first-line therapy in treatment of pancreaticobiliary cancer

**DOI:** 10.3906/sag-2009-115

**Published:** 2021-08-30

**Authors:** Neslihan KAYAHAN, Mustafa KARACA, Hasan SATIŞ, Dilek YAPAR, Ahmet ÖZET

**Affiliations:** 1 Department of Internal Medicine Sciences, Faculty of Medicine, Gülhane Research and Training Hospital, Ankara Turkey; 2 Department of Medical Oncology Sciences, Faculty of Medicine, Antalya Research and Training Hospital, Antalya Turkey; 3 Department of Internal Medicine Sciences, Faculty of Medicine, Gazi University Hospital, Ankara Turkey; 4 Department of Public Health and Bioistatistics Sciences, Faculty of Medicine, Gazi University Hospital, Ankara Turkey; 5 Department of Medical Oncology Sciences, Gazi University Faculty of Medicine Hospital Ankara Turkey

**Keywords:** Pancreatic cancer, folfirinox, gemcitabine-cisplatin, effectiveness, side effect

## Abstract

**Background/aim:**

The purpose of this study was to compare efficacy and safety of a combination chemotherapy regimen consisting of oxaliplatin, irinotecan, fluorouracil, and leucovorin (FOLFIRINOX) and gemcitabine-cisplatin as first-line therapy in patients with pancreatic cancer.

**Materials and methods:**

Pancreaticobiliary cancer patients who had Eastern Cooperative Oncology Group performance status score of 0 or 1 (on a scale of 0 to 5, with higher scores indicating greater severity of illness) were evaluated to receive folfirinox or gemcitabine plus cisplatin. The primary endpoints were progression-free and overall survival time. Safety analysis was also evaluated as secondary measures.

**Results:**

There were 32 patients in the folfirinox group and 36 patients in the gemcitabine-cisplatin group. The median overall survival was 18.1 months (7.5–28.7) in the folfirinox group as compared with 9.7 months (6.5–13) in the gemcitabine-cisplatin group (p = 0.009). Median progression-free survival was 16.2 months (9–23.4) in the folfirinox group and 6.9 months (6.1–7.6) in the gemcitabine-cisplatin group (p = 0.001).

**Conclusion:**

Folfirinox is an option for the first-line treatment of patients with pancreatic cancer and good performance status.

## 1. Introduction

Pancreatic cancer (PC) is one of the leading causes of cancer mortality in the world. Although new therapeutic strategies have been developed; unfortunately, the overall 5-year survival is approximate %5 [1] and the fourth leading cause of deaths caused by cancer in the world [2]. Several treatment modalities including surgery, chemotherapy, and molecular and biological targeted therapy are used for the treatment of pancreatic cancer [3]. Among these modalities, chemotherapy is the treatment option for treating advanced or metastatic PC [4]. Without chemotherapy, life span of patients is reported to be only 2–4 months [5].

A single-agent or certain combination chemotherapy regimens have been shown to prolong survival with tolerable toxicity profiles. Single-agent gemcitabine has been used for local, advanced, and metastatic PC. It has good tolerability and clinical response rate [6,7]. However, despite this potential, PC still had a poor prognosis; thus, new chemotherapy regimens have emerged. Since 2011, folfirinox (oxaliplatin, irinotecan, leucovorin, and fluorouracil combination) regimen has been increasingly used and becoming gold standard in the treatment of pancreatic cancer. In several studies, the overall survivals have ranged from 10 to 32.7 months and progression-free survivals have ranged from 3 to 20 months [8]. On the other hand, side effects including neutropenia, diarrhea, neuropathy, and thrombocytopenia have been seen more often than isolated gemcitabine regimen

Gemcitabine-based regimen is one of the options for treatment of unresectable and metastatic pancreatic cancer. The efficacy and toxicity of this regimen have been studied in several studies. In one of these studies, gemcitabine-cisplatin (Gem-Cis) regimen has better survival results with respect to other gemcitabine combination; however, these results are not statistically meaningful [9]. In terms of toxicity, Gem-Cis combination had unfavorable results [10]; however, in these studies, Gem-Cis combination was not compared to folfirinox in terms of efficacy and safety directly.

In this study, we retrospectively evaluated the safety and efficacy of folfirinox and Gem-Cis combination in unresectable and metastatic pancreatic cancer patients. 

## 2. Materials and methods

Pancreaticobiliary cancer patients who were diagnosed and treated in Gazi University Oncology Department between January 2010 and July 2017 were retrospectively evaluated. Patients’ medical records were investigated and those who were given either only folfirinox or only gemcitabine-cisplatin were selected. Patients who did not come for follow-up, have missing variables, or died before the first follow-up were excluded. Locally advanced or metastatic pancreatic cancer was included. Patients who had Eastern Cooperative Oncology Group performance status score of 0 or 1 were chosen. The primary end-point was survival time at the end of the study. Safety analysis was the secondary end-point. The study was approved by the local ethic committee (approval number: 2017/147).

Oxaliplatin, 85 mg per square meter of the body-surface area; irinotecan, 180 mg per square meter; leucovorin, 400 mg per square meter; and fluorouracil, 400 mg per square meter given as a bolus followed by 2400 mg per square meter given as a 46-h continuous infusion, every 2 weeks were given in the folfironox regimen. Gemcitabine at a dose of 1000 mg per square meter weekly for the 1st and 2nd week, cisplatin at a dose of 100 mg per square meter weekly for the 1st week, every 3 weeks were given in the gemcitabine-cisplatin combination regimen

  Tumor response was evaluated at every 10–12 weeks by computed tomography. In some cases, magnetic resonance imaging (MRI) and positron emission tomography were also used. Tumor response was assessed using computed tomography and graded according to the Response Evaluation Criteria in Solid Tumors (RECIST) version 1.1 [10]. Progression-free survival was defined as starting from the assignment in a clinical trial to disease progression or death from any cause. Overall survival is the duration starting from the time of assignment and continuing until the date of death due to any cause, or until the date of censoring at the last time the subject was known to be alive in intention-to-treat populations.

### 2.1. Statistical analysis

Patients’ demographic data, tumor stage and histopathological characteristics, and chemotherapy-related documented side effects were compared. Continuous data were presented as mean ± SD. Categorical variables were provided as percentages. In univariate analysis, Student’s *t*-test and Mann–Whitney test were used to compare continuous variables while chi-square and Fisher’s exact tests were used for categorical variables. Kaplan–Meier Survival Analysis was used for progression-free survival time and overall survival time estimation. Univariate and multivariate analyses were performed using Cox proportional hazards regression to investigate prognostic factors for progression-free survival (PFS) and overall survival (OS). The variables which showed potential relation with PFS and OS in the univariate analyses (p < 0.2) were further evaluated in the multivariate analyses. A two-sided p-value of 0.05 or less was considered statistically significant. SPSS 15 was used for statistical analysis.

## 3. Results

### 3.1. Baseline characteristics

Pancreaticobiliary cancer was diagnosed in 169 patients. Either folfirinox or Gem-Cis therapy was given to 105 patients. A total of 68 patients were eligible for this study, the remaining 37 patients had missing baseline information or did not attend their first follow-up. Demographic data and baseline characteristics were shown in Table 1. Thirty-two patients received folfirinox and 36 patients received Gem-Cis chemotherapy regimen. The median follow-up time was 13.6 months for the folfirinox group (min/max 4.4 and 45.3 respectively) and for the Gem-Cis groups it was 10.9 months (min/max 5.13 and 46.4 respectively). The number of stage 4 patients was 20 (62.5%) in the folfirinox group and  22 (61.1%) in the gem-cis group. The mean folfirinox given was 8.2 cycle (4–17) and the mean Gem-Cis given was 5.9 (3–14) cycle (p = 0.022). The mean age in the folfirinox group was 50.2, whereas in the Gem-Cis group it was 58.30 (p = 0.27). ECOG performance status was also similar in both groups as shown in Table 1.

**Table 2:** Safety profile of the treatment regimens.

	FOLFIRINOX (n:32)	Gem-Cis (n:36)	p
Anemia (n) (%)	24 (75)	30 (83.3)	0.658
Mild	20 (62.5)	26 (72.2)	0.540
Severe	4 (12.5)	4 (11.1)	0.770
Neutropenia (n) (%)	26 (81.25)	26 (72.2)	0.350
Mild	8 (25)	12 (33.3)	0.590
Severe	18 (56.25)	14 (38.9)	0.224
Thrombocytopenia (n) (%)	19 ( 59.4)	17 (47.2)	0.016
Mild	16 (50)	7 (19.1)	0.080
Severe	3 (9.4)	10 (27.8)	0.060
Liver function tests abn. (n) (%)	11 (34.3)	11(30.4)	0.930
Renal funciton tests abn. (n) (%)	12 (37.3)	6 (16.6)	0.090
Diarhea (n) (%)	3 (9.3)	1 (2.7)	0.520
Neuropathy (n) (%)	0	5 (13.8)	-

The major pathologic tumor type in both treatment groups was adenocarcinoma of the pancreas with 30 (93.8%) patients in the folfirinox group versus 34 (94.4%) patients for the Gem-Cis group. Tumor localizations were also similar for both groups, head of the pancreas were the most frequently seen localization and then corpus and tale followed as the second and third respectively. Stage 4 patients have outnumbered in treatment arms and liver was the primary site for metastasis (Table 1).

### 3.2. Efficacy

**Figure 1:**
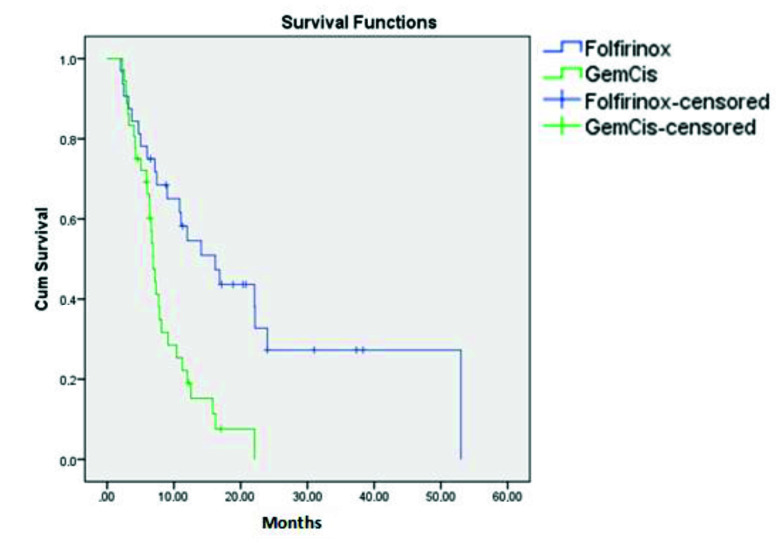
Progression-free survival of the treatment groups.

**Figure 2:**
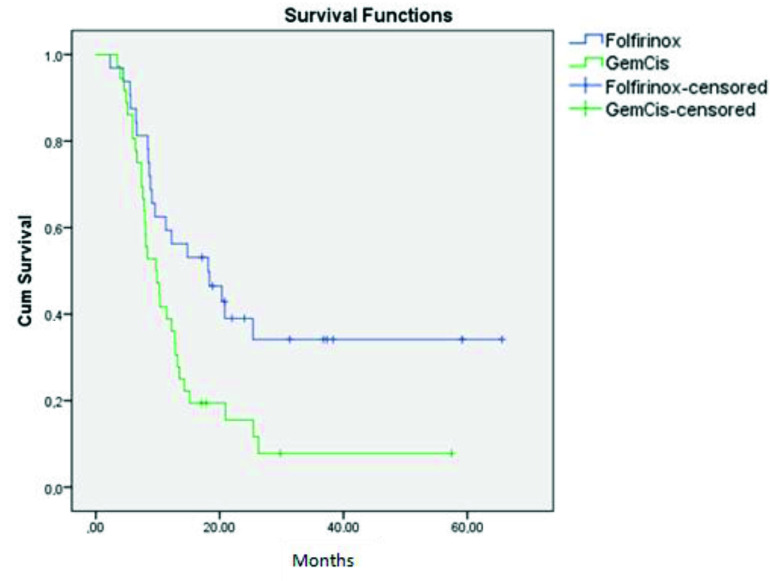
Overall survival of the treatment groups.

Treatment responses and efficacy data were shown below in Figures 1 and 2. At any time 65.6% of the folfirinox patients and 86.1% of the Gem-Cis patients had progression. The median progression-free survival (PFS) time for the folfirinox patients was 16.2 months (9–23.4) whereas for the Gem–Cis patients it was 6.9 months (6.1–7.6) (p = 0.001) (Figure 1) . In subgroup analysis, the number of patients with metastasis was 20 and their median PFS was 16.2 months (9.2–23.2). On the other hand, in the Gem–Cis group there were 22 patients with metastasis and PFS was 6.4 months (5,4–7.4) (p < 0.05). The difference was also obvious in the locally advanced group; for the folfirinox group it was 12 months (3.2–29.3) while for the Gem-Cis group it was 7.1 months (5.9–8.4) (p < 0.05).

At the end of the study, in the folfirinox group 12 patients (37.5%) and in the 

Gem-Cis group 4 patients (11.1%) were still alive. The median overall survival (OS) time for the folfirinox group was 18.1 months (7.5–28.7) and for the Gem–Cis group it was 9.7 months (6.5–13) (p = 0.009) (Figure 2). In subgroup analysis for metastatic group, the median OS in the folfirinox group was 11.3 months ( 5.5–17.4) while it was 10.3 months ( 5.5–15.1) in the Gem–Cis group (p = 0.34). In the locally advanced group, it was 25.4 months ( 22.7–53) and 7.4 months (6–9.7) for the folfirinox and Gem–Cis groups respectively (p = 0.005).

### 3.3. Safety profile

Safety analysis was available for 32 patients in the folfirinox group and for 36 patients in the Gem-Cis group. The results are shown in Table 2. Grade 3-4 neutropenia was more frequently seen in the folfirnox group (56 %vs. 39%, p = 0.224) while grade 3-4 thrombocytopenia was encountered more often in the Gem-Cis group (9.4% vs. 27.8%, p = 0.016). Anemia frequency in any grade was similar for both treatment groups. This was also the same for liver and kidney function test results. The folfirinox group had more liver and kidney dysfunction but it was not statistically significant compared to the Gem-Cis group.

**Table 1:** Demographic and disease characteristics of the study population; ECOG: DM diabetes mellitus, HT: hypertension.

	FOLFIRINOX	Gem-Cis	p
Patient (n)	32	36	
Female (n) (%)	14 (43)	19 (52)	0.309
Age (mean)(std)	57.2 ± 5.22	58.3±4.89	0.456
ECOG (n) (%)			
0	11 (34)	13 (36)	0.245
1	21 (67)	23 (64)	0.227
DM (n) (%)	23 (71.9)	25 (69.4)	0.520
HT(n) (%)	28 (87.5)	24 (66.6)	0.140
Smoking (n) (%)	9 (28.1)	14 (38.9)	0.249
Alcohol (n) (%)	2 (6.3)	3 (8.3)	0.557
Pathology (n) (%)			
Adenocancer	30 (93.8)	34 (94.4)	0.258
Signet Ring Cell	0	1 (2.8)	0.789
Anaplastic	0	1 (2.8)	0.789
Neuroendocrine	2 (6.2)	0	0.612
Tumour Localization (n) (%)			
Head	21 (65.6)	20 (55.6)	0.260
Corpus	3 (9.4)	8 (22.2)	0.345
Tail	3 (9.4)	2 (5.6)	0.482
Duodenum-Biliary Tract	5 (15.6)	6 (16.7)	0.812
Stage (n) (%)			
Stage 2B-3 ( Local Advanced)	12 (37.5)	14 (38.9)	0.553
Stage 4 ( Metastatic)	20 (62.5)	22 (61.1)	0.512

The number of patients with diarrhea which resulted in hospitalization was 3 in the folfirinox group, whereas it was only 1 in the Gem-Cis group (p = 0.52). There were not any patients having a neurotoxicity in the folfirnox group; however, in the Gem-Cis group, there were 5 patients who suffered from severe neuropathic complaints and had abnormal EMG consistent with neuropathic dysfunction.

As a result of side effects, the number of patients having chemotherapy dose reduction was 4 in the folfirinox group and 5 in the Gem-Cis group. On the other hand, treatment was terminated in 2 patients in the folfirinox group and in 5 patients in the Gem-Cis group (p > 0.05).

## 4. Discussion

Prognosis of pancreatic cancer is still far from being satisfactory, especially for metastatic cases. Several chemotherapy protocols have been used and gemcitabine-based protocols historically have had an important role in treatment. By combining with other chemotherapeutics, it was thought that better response rates could be achieved. However, as this study shows, the folfirinox regimen was superior to the gemcitabine–cisplatin combination. The safety analysis of both treatment regimens was similar; thus, folfirinox must be used as a first-line therapy in the treatment of pancreaticobiliary cancer.

The gemcitabine–cisplatin combination was previously compared to isolated gemcitabine treatment in a phase 3 study. In combination regimen, progression-free survival and the overall response rate was better but overall survival rate and safety profile were not different between groups [11]. Contrarily, this combination resulted in more hematological side effects in another study [12]. In a review comparing gemcitabine-based regimens, in subgroup analysis, gemcitabine-cisplatin combination had an advantage for survival but it did not reach statistical significance [9]. For the tumor response rate, gem-cis regimen had a better partial and overall response rate. Moreover, disease progression rate was worse in isolated gemcitabine group [12–15]. On the other hand, the folfirinox regimen had a strong impact on overall survival and progression-free survival compared to isolated gemcitabine treatment [16–18].

In literature, there are not any studies directly comparing these two regimens, to our knowledge. Folfirnox protocol had better survival ratios but also had more side effects as compared to isolated gemcitabine protocol. In this study, the folfinox group had more neutropenia, diarrhea thrombocytopenia, and sensorial neuropathy [18]. In a metaanalysis comparing all chemotherapy protocols used in pancreatic cancer were evaluated in terms of toxicity and efficacy. Although there were not any direct comparison, folfirinox had the best short- and long-term efficacy among the 12 chemotherapy regimens. On the other hand, folfirinox and Gemcitabine + Pemetrexed regimens had a relatively higher incidence of toxicity than other regimens [19]. Another metaanalysis comparing toxicity profiles of these regimens, Gemcitabine + Cisplatin and folfirinox regimens exhibited the highest incidence rates of neutropenia [20].

 In this study, severe neutropenia and diarrhea were more frequently seen in the folfirinox group but it did not reach statistical significance. Contrarily, severe thrombocytopenia was more prevalent in the gemcitabine group (p = 0.069). All patients with the diagnosis of neuropathy belonged to the gemcitabine group. It may be as a result of the shorter follow-up period for the folfirinox group and also this should be cautiously interpreted with the consideration of additive cisplatin toxicity that might have an impact on these increased side effects.

 Survival ratios were consistent with the literature. In a review, 11 studies were included and the mean overall survival was 24.2 months (10–32) and the mean progression-free survival was 15 months ( 3–20) [8]. Moreover, folfirinox protocol survival ratios were significantly better than those of the gemcitabine group in locally advanced patients. However, the difference diminished in the metastatic group. Increased tumor burden of the disease that rendered the difference irrelevant might be the explanation 

 This study has some limitations. It is retrospective, single-center, and nonrandomized and this may cause selection bias and confound. Moreover, safety profiles data in our analysis may have been missed due to a lack of identification of adverse events as a result of being retrospective data. Moreover, our study population was homogeneous as most patients had good PS, which might have affected the tolerability of the regimens. 

 In conclusion, folfirinox is a better option for locally advanced and metastatic pancreaticobiliary carcinoma treatment than gemcitabine–cisplatin combination and can be used as first-line chemotherapy in the real-world setting. Toxicity profile should be kept in mind, especially hematological and gastrointestinal toxicity, which can cause severe morbidity and mortality and be managed by decreasing or modifying drug dosage.

## Funding

The authors received no financial support for the research, authorship, and/or publication of this article.

## Ethical approval

This study was approved by the ethical committee of Gazi University (approval number 2017/147).
